# interPopula: a Python API to access the HapMap Project dataset

**DOI:** 10.1186/1471-2105-11-S12-S10

**Published:** 2010-12-21

**Authors:** Tiago Antao

**Affiliations:** 1 Liverpool School of Tropical Medicine, L3 5QA, Liverpool, UK

## Abstract

**Background:**

The HapMap project is a publicly available catalogue of common genetic variants that occur in humans, currently including several million SNPs across 1115 individuals spanning 11 different populations. This important database does not provide any programmatic access to the dataset, furthermore no standard relational database interface is provided.

**Results:**

interPopula is a Python API to access the HapMap dataset. interPopula provides integration facilities with both the Python ecology of software (e.g. Biopython and matplotlib) and other relevant human population datasets (e.g. Ensembl gene annotation and UCSC Known Genes). A set of guidelines and code examples to address possible inconsistencies across heterogeneous data sources is also provided.

**Conclusions:**

interPopula is a straightforward and flexible Python API that facilitates the construction of scripts and applications that require access to the HapMap dataset.

## Background

The HapMap project [1] (http://hapmap.ncbi.nlm.nih.gov/) is an effort to identify and catalogue genetic similarities and differences in humans. The project makes information available on single nucleotide polymorphisms (SNPs), and it more recently added information on copy number variation (CNV). HapMap phase 3 includes data on 1115 individuals (around 1.5 million SNPs per individual) spanning 11 populations while phase 2 included only 4 populations (270 individuals) but more than 3.5 million SNPs per individual. This dataset can be useful in a multitude of situations from finding genes that affect human health to evolutionary research about the human species or for genome-wide association studies. All of the information generated is released into the public domain and can be downloaded with minimal constraints. The HapMap project provides access to the data in bulk form (via FTP download), a web interface [2] which includes a genome browser [3] and the data mining application HapMart based on Biomart [4]. Programmatic and relational database interfaces are not offered though some API support is implemented by external parties such as a generic Perl API for variation datasets in Ensembl [5], BioPerl's Bio::PopGen module [6] or the GGtools package [7] for R/Bioconductor. Most existing libraries support only a subset of features (e.g. parsing of HapMap file formats or creating a local database) making the construction of scripts and applications more complex as basic data manipulation functionality must be built as least partially. Furthermore, there is no known Python library supporting HapMap data.

## Implementation

interPopula provides a Python API to access the HapMap dataset. Interfaces to all HapMap phases are supported including phase 2 data with fewer populations but more SNPs genotyped per individual and phase 3 covering more populations. interPopula provides access to frequency, genotype, linkage disequilibrium and phasing datasets. The recent CNV dataset is also supported along with family relationships for the 5 populations where sampling was performed for family trios (mother, father and one offspring).

Support for annotation information that is commonly needed to process HapMap data is also provided through an API to both the UCSC Known Genes dataset [8] from the UCSC genome browser database [9] and the Ensembl gene annotation database [10].

The API was constructed according to the following design guidelines:

1. The API is straightforward and self-contained. The core API requires only a Python interpreter, has no extra dependencies and minimal administrative overhead.

2. Downloaded data is stored on an SQL database for faster access. All data is stored using sqlite [11] which is natively supported in Python thus lowering the maintenance costs of the system. interPopula can also be connected to enterprise-grade databases which support multiple users, concurrent usage and large datasets for which the standard sqlite backend might not be enough (a PostgreSQL example is provided).

3. Data management (i.e. downloading from the HapMap site and local database construction) is fully automated: the required data subset is downloaded on demand only once and stored locally, reducing the load on both the client and server.

4. While SQL interfaces are made available from both the UCSC and Ensembl projects for their annotation databases, interPopula uses the same implementation strategy for the HapMap dataset: files are intelligently downloaded and locally stored. This provides a consistent interface to these two datasets which provide important annotation information frequently used to process HapMap data.

5. The framework is extensible and designed to be easily integrated with other Python tools and external databases. The web site provides several examples of integration with standard tools used in Python for bioinformatics such as Biopython [12], NumPy [13] and matplotlib [14].

6. Integration with Biopython allows for access to the Entrez SNP database and the population genetics tools supported by Biopython such as Genepop [15] allowing automated analysis of datasets.

7. Facilities to export HapMap data to Genepop format are provided enabling (non-automated) analysis of the HapMap dataset with the plethora of population genetics software which support this format. Data export can also be use to initialize population genetics simulators like the Python-based simuPOP [16] allowing computational simulations to be initialised with real datasets.

8. A large set of scripts is included, serving both as utilities to analyse the data, as well as examples of database and external tool integration. Currently we provide examples of integration with Entrez databases (nucleotide and SNP), the Genepop population genetics suite and charting libraries.

9. A set of guidelines and scripts was developed in order to facilitate a consistent view across heterogeneous databases. HapMap, Ensembl, UCSC Known Gene and the Entrez databases might not be fully consistent among themselves and, if care is not taken, database integration efforts might lead to erroneous results. The main pitfall is the usage of different NCBI reference builds across different databases, most notably HapMap is still based on build 36 whereas other databases either support multiple builds or only the most recent build 37.

10. A robust open-source software development process is put in place: a full public web based platform (hosted on Launchpad) is used to maintain the code infrastructure and unit tests approach 100% coverage.

## Results

interPopula can be used to create a wide range of applications and scripts based on the HapMap dataset. The most commonly expected usage pattern will be for genome wide association studies, though the example presented here will be of a different nature.

As an example of usage, we present a population comparison of all the genotyped SNPs for a gene. We will plot the *F_st_* statistic for all Lactase SNPs between two HapMap populations: Utah residents with Northern and Western European ancestry (CEU) and Yoruban in Ibadan, Nigeria (YRI). These populations are known to differ in their tolerance to lactose [17]. This example uses genotype information from HapMap and also demonstrates the integration facilities with UCSC Known Genes (to retrieve gene position and exon data), matplotlib (used for plotting), Biopython and Genepop (used to calculate *F_st_*).

This example, which is quite complex in terms of integration between several databases and tools can be broken down into the following steps:

1. Load the Known Genes database. The version pertaining build 36 should be loaded to assure consistency with HapMap.

2. Determine relevant information about Lactase from Known Genes. The following information is needed: The chromosome on which it is located, the start and end positions in the chromosome and all exon positions.

3. Load HapMap genotype information for the CEU and YRI populations for the relevant chromosome.

4. Retrieve all the HapMap SNP ids between the start and end positions in the chromosome.

5. Export a Genepop formatted file with two populations including all HapMap SNPs for Lactase.

6. Call the Genepop application via Biopython to calculate the *F_st_* for all markers.

7. Plot the calculated *F_st_s* along with the exon positions.

**Figure 1:**
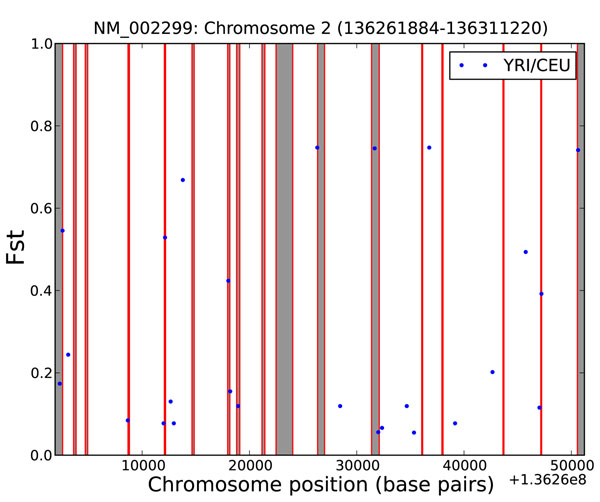
***F_st_* for Lactase between 2 HapMap populations.***F_st_* between CEU and YRI populations for all Lactase SNPs on the HapMap database. The X-axis reports the position on chromosome 2 (the value on the lower right is the absolute offset from the beginning of the chromosome), the Y-axis the *F_st_* value. The dots represent the *F_st_* values for existing SNPs on the HapMap database. The red boxes represent exon positions. To construct this chart HapMap frequency data and the UCSC Known Genes database were consulted. Biopython and Genepop were used to compute the *F_st_* statistic.

The result of this example is shown in Figure 1. The X-axis reports the position along chromosome 2, *F_st_*in on the Y-axis, the dots represent the *F_st_* values for existing SNPs on the HapMap database and the red boxes are the exon positions (17 in the case of Lactase). Interpreting the results of this specific application of interPopula is beyond the scope of this manuscript but at least two different interpretations are possible: (i) SNPs where *F_st_* is above approximately 0.45 are candidates for positive selection (as around 95% of *F_st_*values for humans are below 0.45 [18]) or (ii) the *F_st_* statistic is noisy when applied to a single marker [19] . The above example was constructed using the UCSC Known Genes database but the programmer can alternatively use the Ensembl gene annotation database instead.

This example (script IGFstGene.py in the distribution), along with more than 20 others including data export, connection to enterprise-grade databases, analysis of the distribution of the number of exons per gene, the distribution of genes per chromosome are made available with interPopula.

**Figure 2:**
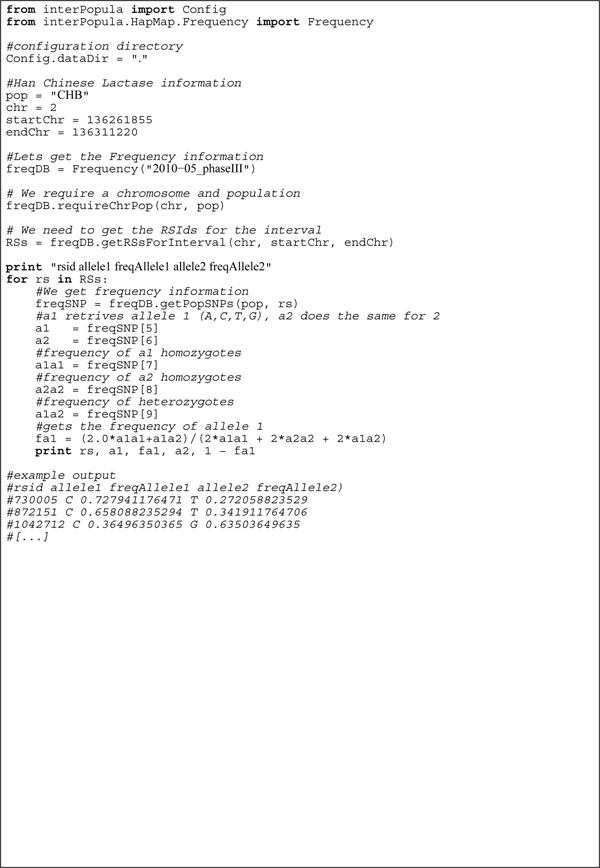
**Example code to print the frequency of HapMap SNPs**. This example describes how to consult the HapMap frequency database to retrieve the allele frequencies for a set of SNPs in a section of a chromosome.

In order to illustrate interPopula’s basic API, Figure 2 shows a commented script which provides useful functionality. In this example the HapMap frequency database is consulted to report the frequency of both alleles for each SNP within a certain chromosome interval. The code example is less than one page in length and there are only 4 API calls to achieve the complete functionality. This is one case illustrating the ease of use of the API. All scripts provided with interPopula are documented to the level of the example presented and automated documentation covering the full API is extracted from the source using epydoc (http://epydoc.sourceforge.net/).

The part of the API devoted to both UCSC Known Genes and the Ensembl gene annotation database can be used stand-alone to access both databases, i.e., it can be used for application and scripts that have no relationship with the HapMap data. interPopula’s UCSC and Ensembl APIs can be used to access also non-human data as genome annotations are available for other species. This is especially useful with the Ensembl dataset as it makes available gene annotation information for many other species. Users should note that the quality of the datasets for other species varies as more effort is put in the curation of human data (e.g. while for humans the chromosome information is normally the chromosome number, for cats - *Felis catus* - it is mostly scaffold data). Stand-alone example script examples are provided for both datasets.

Future development efforts for interPopula will focus on supporting large datasets. As sequencing costs continue to decrease and the sequencing of complete genomes becomes commonplace it is clear that the backend infrastructure will have to be redesigned to support the large amounts of data generated by such efforts. In this context, supporting the 1000 genomes project [20] is a natural extension for interPopula as many of the samples used in this project come from the HapMap dataset. While the API for UCSC and Ensembl extensions provides access to other species data, the main focus of interPopula will remain providing robust and well-maintained APIs for publicly available human genomic datasets which lack a standardized Python API or relational interface.

## Conclusions

interPopula is a flexible, straightforward Python API to the HapMap project. It strives to integrate with both common Python bioinformatics and scientific libraries and other genomic databases that are commonly used in conjunction with the HapMap dataset. interPopula makes HapMap data processing possible inside Python, thus opening the possibility for the development of a plethora of interesting applications and scripts that make use of this important resource for human population genomics studies.

## List of abbreviations used

API - Application Programming Interface; CEU - HapMap population sample comprising Utah residents with Northern and Western European ancestry; CNV - Copy Number Variation; SNP - Single Nucleotide Polymorphism; SQL - Structured Query Language; UCSC - University of California, Santa Cruz; YRI -HapMap population sample comprising Yoruban in Ibadan, Nigeria

## Competing interests

The author declares that he has no competing interests.

## Availability and requirements

**Project name** interPopula

**Project home page**http://popgen.eu/soft/interPop/. Development site: https://launchpad.net/interpopula

**Operating systems** Platform independent

**Programming language** Python

**Other requirements** Optionally NumPy, Biopython, Genepop and matplotlib

**License** GNU GPL version 3

**Any restrictions to use by non-academics** None
